# The Relationship Between Predisposing Risk Factors and COVID-19: An Observational Study

**DOI:** 10.7759/cureus.75042

**Published:** 2024-12-03

**Authors:** Ramnivas Vishnoi, Manish Gaba, Naveen Kumar, Ankita Pandey, Arun Dewan

**Affiliations:** 1 Internal Medicine, Max Smart Super Speciality Hospital, New Delhi, IND

**Keywords:** comorbidities, covid-19, india, risk factor, sociodemographic variables

## Abstract

Background

Numerous risk factors have been identified for developing severe COVID-19, including sociodemographic variables and concomitant diseases. Individuals with underlying comorbidities such as diabetes, hypertension, asthma, and coronary artery disease are at a greater risk of severe illness and death. This study aimed to observe the association between risk factors and the severity of COVID-19.

Methodology

A single-center, hospital-based, prospective, observational study was conducted at Max Smart Super Speciality Hospital in Saket, Delhi from October 2020 to December 2021. A total of 1,454 patients admitted under our care in the Department of Internal Medicine were included in this study. Patients were divided into the following three groups: patients without comorbidities, patients with a single comorbidity, and patients with multiple comorbidities. The risk factors under evaluation were age >50 years, obesity, diabetes, hypertension, chronic kidney disease (CKD), heart disease, chronic liver disease (CLD), and immunocompromised status (human immunodeficiency virus, post-transplant, malignancy undergoing chemotherapy).

Results

In this study, 28.1% (n = 408) of patients did not have comorbidities, 30.1% (n = 438) of patients had a single comorbidity, and 41.8% (n = 608) of patients had multiple comorbidities. Regarding risk factors, 62% (n = 872) of patients were aged >50 years, 7.4% (n = 108) were obese, 30.7% (n = 447) had diabetes, 33% (n = 480) were hypertensive, 1.2% (n = 18) had CKD, 6.8% (n = 99) had heart disease, 0.3% (n = 4) had CLD, and 5.5% (n = 80) were immunocompromised. A statistically significant association was found between increasing age and worsening severity of COVID-19 (p = 0.0001), male gender (p = 0.0001), presence of comorbidities, including diabetes, hypertension, obesity, CKD, CLD, heart disease (p = 0.0001). Patients in the immunocompromised group did not have a statistically significant association with disease severity. A statistically significant association was found between mortality and severity of COVID-19. Overall, 16.7% (n = 48) of the patients in the no comorbidity group, 35.4% (n = 102) in the single comorbidity group, and 47.9% (n = 138) in the multiple comorbidity group (p = 0.0001) presented with severe disease on admission.

Conclusions

The study shows that the severity of the disease increased as the number of risk factors increased. This information can help us take early and active measures in these groups of patients with multiple comorbid illnesses.

## Introduction

The clinical spectrum of COVID-19 is extremely variable, ranging from asymptomatic infection and mild upper respiratory tract illness to severe viral pneumonia with respiratory failure and multiorgan dysfunction. Numerous risk factors have been identified for developing severe COVID-19, including sociodemographic variables and concomitant diseases. Individuals with underlying comorbidities such as diabetes, hypertension, asthma, and coronary artery disease are at a greater risk of severe illness and death [1-3]. However, the majority of preceding studies, particularly those published early in the pandemic, did not use multivariable adjustment to uncover independent risk variables. Moreover, just a few assessed a range of illness outcomes, including hospitalization, mechanical ventilation, and death. The majority of earlier research was limited to a local or regional scale rather than a national level. Finally, the majority of studies did not compare the differences in patients with different severity to evaluate the additional risk associated with COVID-19 infection.

This study aimed to observe the association between risk factors and the severity of COVID-19. The study aimed to identify patients with risk factors, clinically assess disease severity, and observe the risk of severe adverse outcomes by stratifying according to the number and type of comorbidities, unraveling the sub-population with poor prognosis.

## Materials and methods

Study goal

The study aimed to observe any association between risk factors (none vs. one risk factor vs. multiple risk factors) and disease severity on presentation. It also aimed to observe any association between risk factors and the disease course, including the need for non-invasive ventilation, invasive ventilation, intensive care, or mortality.

Study design

A single-center, hospital-based, prospective, observational study was conducted at Max Smart Super Specialty Hospital in Saket, Delhi from October 2020 to December 2021. A total of 1,454 patients admitted under our care in the Department of Internal Medicine were included in the study. Patients were divided into the following three groups: patients without comorbidities, patients with a single comorbidity, and patients with multiple comorbidities. The various risk factors studied include diabetes (type 1 and type 2), hypertension, obesity (body mass index (BMI) >30 kg/m^2^), chronic kidney disease (CKD), heart disease (coronary artery disease, cardiomyopathies), chronic liver disease (CLD), immunocompromised status (long-term steroids, malignancy on chemotherapy, transplant recipients, connective tissue disorder on disease-modifying agents, myasthenia on immunosuppressants, human immunodeficiency virus), and age >50 years. The inclusion criteria consisted of (1) a positive reverse transcriptase polymerase chain reaction test for COVID-19 and (2) age >18 years. The exclusion criteria consisted of (1) a history of smoking, (2) a history of lung diseases (asthma, chronic obstructive airway disease), (3) age <18 years, and (4) patients with acute kidney injury.

Sampling

The objective of the study was to determine the prevalence of the various risk factors in COVID-19. The study by Guan et al. was used to calculate sample size [4]. To estimate this within nearly 1/10th of this rate with a confidence level of 95%, the sample size was calculated to be 1,349. Every consecutive patient, fulfilling the inclusion and exclusion criteria, was enrolled to achieve the sample size in the stipulated duration, hence convenience sampling was utilized.

Data collection

In this study, clinical severity was assessed as per the Ministry of Health and Family Welfare (MOHFW), India guidelines. Patients were divided into the following three groups: no comorbidities, single comorbidity, and multiple comorbidities (Table 1) [5]. Data collection was done by personal interviews, including demographic information, clinical assessment, and review of laboratory investigations. Based on this, patients were classified as having mild, moderate, or severe disease.

**Table 1:** Clinical severity and assessment parameters according to MOHFW guidelines [5]. MOHFW: Ministry of Health and Family Welfare; SpO_2_: oxygen saturation; ARDS: acute respiratory distress syndrome; PaO_2_: partial pressure of oxygen; FiO_2_: fraction of inspired oxygen

Severity	Details
Mild	Patients with uncomplicated upper respiratory tract infections may have mild symptoms such as fever, cough, sore throat, nasal congestion, malaise, and headache without evidence of breathlessness or hypoxia (normal saturation)
Moderate	Pneumonia with no signs of severe disease. Presence of clinical features of dyspnea and/or hypoxia, fever, cough, SpO_2_ <94% (range = 90-94%) on room air, and respiratory rate of more or equal to 24 breaths/minute
Severe pneumonia	Clinical signs of pneumonia plus one of the following: respiratory rate >30 breaths/min, severe respiratory distress, and SpO_2_ <90% on room air
ARDS	New or worsening respiratory symptoms within one week of known clinical insult. Chest imaging: bilateral opacities, not fully explained by effusions, lobar or lung collapse, or nodules. The origin of pulmonary infiltrates is not fully explained by cardiac failure or fluid overload. Oxygenation impairment. Mild ARDS: PaO_2_/FiO_2_ = 200 mmHg to ≤300 mmHg. Moderate ARDS: PaO_2_/FiO_2_ = 100 mmHg to ≤200 mmHg. Severe ARDS: PaO_2_/FiO_2_ = ≤100 mmHg

Data analysis

Data were recorded into Microsoft® Excel workbook 2019 and exported for statistical evaluation into SPSS version 21.0 (IBM Corp., Armonk, NY, USA)). Data were presented as frequency, percentage, mean, and standard deviation. The association between two variables was measured using the chi-square test. P-values <0.05 were considered significant.

## Results

In this study, 28.1% (n = 408) of patients did not have comorbidities, 30.1% (n = 438) of patients had a single comorbidity, and 41.8% (n = 608) of patients had multiple comorbidities. Regarding risk factors, 62% (n = 872) of patients were aged >50 years, 7.4% (n = 108) were obese, 30.7% (n = 447) had diabetes, 33% (n = 480) had hypertension, 1.2% (n = 18) had CKD, 6.8% (n = 99) had heart disease, 0.3% (n = 4) had CLD, and 5.5% (n = 80) were immunocompromised.

In our study, the mean age of the study participants was 53.5 years, with an SD of 15.2. Of the 1,454 participants, 872 (60%) were above the age of 50 years, and 986 (67.8%) were males. The most common symptoms were fever (96.8%), followed by dry cough (71.5%) and fatigue (33%) (Table 2).

**Table 2:** Distribution of study participants according to symptoms.

Symptoms	Frequency	Percent
Fever	1,408	96.8
Conjunctival congestion	10	0.7
Nasal congestion	61	4.2
Headache	329	22.6
Dry cough	1,039	71.5
Pharyngodynia	15	1.0
Productive cough	28	1.9
Fatigue	480	33.0
Hemoptysis	10	0.7
Shortness of breath	374	25.7
Nausea vomiting	25	1.7
Diarrhoea/Myalgia/Arthralgia	79	5.4

Oxygen support was required in 29.8% of our participants (Table 3).

**Table 3:** Distribution of study participants according to oxygen requirement.

Oxygen requirement	Frequency	Percent
Yes	433	29.8
No	1,021	70.1
Total	1,284	100

Of the 1,454 participants, 408 (28.1%) did not have any comorbidity, 438 (30.1%) had a single comorbidity, and 608 (41.8%) had more than one comorbidity (Table 4).

**Table 4:** Distribution of study participants according to comorbidity.

Comorbidity	Frequency	Percent
No	408	28.1
Single comorbidity	438	30.1
Multiple comorbidities	608	41.8
Total	1,454	100.0

Hypertension (33%) was the most common comorbidity, followed by diabetes (30.7%) (Table 5).

**Table 5:** Distribution of study participants according to comorbidities. DM: diabetes mellitus; HTN: hypertension; BMI: body mass index; CKD: chronic kidney disease; CLD: chronic liver disease

Comorbidity	Frequency	Percent
DM	447	30.7
HTN	480	33.0
Obesity (BMI >25 kg/m^2^)	108	7.4
CKD	18	1.2
Heart disease	99	6.8
CLD	4	0.3
Immunosuppressed	80	5.5

The severity of illness in most of the patients was mild (651, 44.8%), followed by 515 (35.4%) patients having moderate and 288 (19.8%) having severe COVID-19 (Table 6).

**Table 6:** Distribution of study participants according to the severity on admission.

Severity	Frequency	Percent
Mild	651	44.8
Moderate	515	35.4
Severe	288	19.8
Total	1,454	100.0

Of 1,454 patients, intensive care unit (ICU) admission was needed in 67 (4.6%) cases, and 35 (2.4%) required invasive ventilation. In our study, the mean duration of hospital stay among study participants was 8.1 days with an SD of 3.9 days. Overall, 1.0% of patients died due to COVID-19. A statistically significant association was found between increasing age and worsening severity of COVID-19 (Table 7).

**Table 7:** Association of severity with age.

Age	Severity						P-value
	Mild		Moderate		Severe		
	Count	%	Count	%	Count	%	
18–50 years	312	47.9%	176	34.2%	94	32.6%	0.0001
>50 years	339	52.1%	339	65.8%	194	67.4%	
Total	651	100.0%	515	100.0%	288	100.0%	

A statistically significant association was found between gender and the severity of COVID-19, with the disease being more severe in male patients (Table 8).

**Table 8:** Association of severity with gender.

Gender	Severity						P-value
	Mild		Moderate		Severe		
	Count	%	Count	%	Count	%	
Female	229	35.2%	154	29.9%	85	29.5%	0.0001
Male	422	64.8%	361	70.1%	203	70.5%	
Total	651	100.0%	515	100.0%	288	100.0%	

In the present study, dry cough, nasal congestion, and shortness of breath were found to be statistically higher in frequency in severe COVID-19 patients (Table 9).

**Table 9:** Association of severity with symptoms. SOB: shortness of breath

Symptoms	Severity						P-value
	Mild		Moderate		Severe		
	Count	%	Count	%	Count	%	
Fever	637	97.8%	491	95.3%	280	97.2%	0.055
Conjunctival congestion	2	0.3%	6	1.2%	2	0.7%	0.212
Nasal congestion	35	5.4%	11	2.1%	15	5.2%	0.015
Headache	137	21.0%	133	25.8%	59	20.5%	0.096
Dry cough	436	67.0%	379	73.6%	224	77.8%	0.001
Pharyngodynia	7	1.1%	3	0.6%	5	1.7%	0.297
Productive cough	11	1.7%	9	1.7%	8	2.8%	0.500
Fatigue	204	31.3%	177	34.4%	99	34.4%	0.473
Hemoptysis	3	0.5%	6	1.2%	1	0.3%	0.260
SOB	165	25.3%	105	20.4%	104	36.1%	0.0001
Nausea vomiting	4	0.6%	13	2.5%	8	2.8%	0.014
Diarrhoea/Myalgia/Arthralgia	30	4.6%	30	5.8%	19	6.6%	0.412

A statistically significant association was found between comorbidity and the severity of COVID-19 (Table 10).

**Table 10:** Association of severity with comorbidity.

Comorbidity	Severity						P-value
	Mild		Moderate		Severe		
	Count	%	Count	%	Count	%	
No	319	49.0%	41	8.0%	48	16.7%	0.0001
Single	163	25.0%	173	33.6%	102	35.4%	
Multiple	169	26.0%	301	58.4%	138	47.9%	
Total	651	100.0%	515	100.0%	288	100.0%	

All comorbidities except the immunocompromised state were found to be statistically high in frequency among severe COVID-19 patients (Table 11).

**Table 11:** Association of severity with comorbidities. DM: diabetes mellitus; HTN: hypertension; CKD: chronic kidney disease; CLD: chronic liver disease

Comorbidities	Severity						P-value
	Mild		Moderate		Severe		
	Count	%	Count	%	Count	%	0.0001
DM	114	17.5%	224	43.5%	109	37.8%	0.0001
HTN	128	19.7%	228	44.3%	124	43.1%	0.001
Obesity	31	4.8%	45	8.7%	32	11.1%	0.0001
CKD	0	0.0%	16	3.1%	2	0.7%	0.0001
CLD	1	0.2%	0	0.0%	3	1.0%	0.019
Heart disease	21	3.2%	60	11.7%	18	6.3%	0.0001
Immunocompromised	26	4.0%	36	7.0%	18	6.3%	0.069

A statistically significant association was found between oxygen requirement and the severity of COVID-19. Oxygen requirement increased as severity increased (Table 12).

**Table 12:** Association of severity with oxygen requirement.

Oxygen requirement	Severity						P-value
	Mild		Moderate		Severe		
	Count	%	Count	%	Count	%	
No	547	84.0%	365	70.9%	109	37.8%	0.0001
Yes	104	16.0%	150	29.1%	179	62.2%	
Total	651	100.0%	515	100.0%	288	100.0%	

A statistically significant association was found between ICU/high dependency unit (HDU) admission and the severity of COVID-19. Admission increased as the severity worsened (Table 13).

**Table 13:** Association of severity with ICU/HDU admission. ICU: intensive care unit; HDU: high-dependency unit

Admission	Severity						P-value
	Mild		Moderate		Severe		
	Count	%	Count	%	Count	%	
No	636	97.7%	504	97.9%	247	85.8%	0.0001
Yes	15	2.3%	11	2.1%	41	14.2%	
Total	651	100.0%	515	100.0%	288	100.0%	

A statistically significant association was found between invasive ventilation requirements and the severity of COVID-19 (Table 14).

**Table 14:** Association of severity with invasive ventilation.

Invasive ventilation	Severity						P-value
	Mild		Moderate		Severe		
	Count	%	Count	%	Count	%	
No	642	98.6%	506	98.3%	271	94.1%	0.0001
Yes	9	1.4%	9	1.7%	17	5.9%	
Total	651	100.0%	515	100.0%	288	100.0%	

 A statistically significant association was found between mortality and severity of COVID-19 (Table 15).

**Table 15:** Association of severity with mortality.

Mortality	Severity						P-value
	Mild		Moderate		Severe		
	Count	%	Count	%	Count	%	
No	649	99.7%	511	99.2%	279	96.9%	0.0001
Yes	2	0.3%	4	0.8%	9	3.1%	
Total	651	100.0%	515	100.0%	288	100.0%	

## Discussion

Many studies have been conducted to emphasize the details of the clinical and virological course of COVID-19 infection. Few indicated that individuals with older age and comorbidities such as diabetes and hypertension were more prone to COVID-19 and had a higher risk of mortality. The present study was a hospital-based, observational study conducted among patients diagnosed with COVID-19 by RT-PCR, who were admitted or presented to medicine departments of the Max Super Specialty Hospital, Saket, New Delhi. The study aimed to evaluate the association of risk factors with disease severity and its effect on progression during the stay in hospital. Following the consecutive sampling method, a total of 1,454 study participants were included.

In our study, the mean age of the study participants was 53.5 years with an SD of 15.2, and 872 patients (60%) were above the age of 50. The majority of participants were male (986, 67.8%). A higher incidence rate of COVID-19 in men might be due to higher social interactions in work and public places. Similar results were reported by Mohan et al, who reported a mean age of 40.1 ± 13.1 years, with 93.1% of participants being male [6]. Teklu et al. reported that most patients were in the age group of 31-50 years, and 72.7% were male participants [7].

In our study, of the 1,454 participants, the most common symptoms were fever (96.8%), followed by dry cough (71.5%), and fatigue (33%). Other symptoms include headache (22.6%) and shortness of breath (24.7%). In the study by Mohan et al., a significant proportion of patients had no symptoms (n = 64, 44.4%); among the symptomatic, cough (34.7%) was the most common, followed by fever (17.4%), and nasal symptoms (2.15%) [6]. Teklu et al. reported cough, headache, and fever as the most frequent symptoms [7].

Of the 1,454 participants, 408 (28.1%) did not have any comorbidity, 438 (30.1%) had a single comorbidity, and 608 (41.8%) had more than one comorbidity. The most common comorbidity was hypertension (33%), followed by diabetes (30.7%). In the study by Mohan et al., comorbidities were present in 23 (15.9%) patients, with diabetes mellitus (n = 16, 11.1%) being the most common [6]. Guan et al. reported that 399 (25.1%) of the 1,590 COVID-19 patients in the initial cohort had at least one comorbidity, while 130 (8.2%) had two or more comorbidities [8]. The most common comorbidities in all COVID-19 patients were hypertension (16.9%), diabetes (8.2%), cardiovascular disease (3.7%), and CKD (1.3%). In the study by Chen et al., comorbidities were present in 51% (50/99) of COVID-19 patients, including cardiovascular or cerebrovascular diseases (40.4%), diabetes (12%), digestive system diseases (11%) and malignant tumors (0.01%) [9].

In our study, oxygen support was required in 28.1% of participants, 67 (4.6%) needed ICU admission, and 35 (2.4%) required invasive ventilation. The mean duration of hospital stay among study participants was 8.1 days, with an SD of 3.9. In the study by Mohan et al., only 3.5% of patients required oxygen supplementation, 2.8% of patients had severe disease requiring intensive care, and mortality occurred in 1.4% of patients [6].

In our study, male gender, older age, comorbidity, hospital admission, ICU admission, oxygen requirement, and mortality were found to be associated with the severity of COVID-19. According to Bai et al., having an underlying disease increases the likelihood of COVID-19 [10]. According to a previous case series published in 2020 by Xie et al., the presence of underlying diseases and subsequent hospitalization in the ICU, as well as death, are interrelated in COVID-19 patients [11]. In a study by Zhao et al., 38.10% of cases and 21.43% of controls had comorbidities [12]. In 2020, Yang et al. conducted a meta-analysis to determine the prevalence of comorbidities in COVID-19 patients [13]. The most prevalent comorbidities were hypertension (21.1%, 95% confidence interval (CI) 13.0-27.2%) and diabetes (9.7%, 95% CI = 7.2-12.2%), followed by cardiovascular disease (8.4%, 95% CI = 3.8-13.8%) and respiratory system disease (1.5%, 95% CI = 0.9-2.1%). They inferred that hypertension, respiratory system disease, and cardiovascular disease are risk factors for severe disease [13].

Chronic diseases such as hypertension, diabetes, respiratory system disease, and cardiovascular disease and their risk factors may be associated with the pathogenesis of COVID-19. Odegaard et al. proposed that chronic diseases share some common characteristics with infectious diseases, such as a pro-inflammatory state causing a weakening of the natural immune response [14]. Furthermore, Dooley et al. reported that metabolic diseases may lead to weakened immune function by impairing macrophage and lymphocyte function, making people more vulnerable to the disease complications of COVID-19 [15]. Hong et al. in Korea conducted a prospective case-control study on seasonal influenza in 2014 to identify the factors that increase the risk of flu and its negative consequences. Their findings revealed that chronic cardiovascular disease and diabetes were linked to increased influenza complications. Diabetes as an independent risk factor increased the risk of severe seasonal influenza by more than 3.5 times [16]. Furthermore, Alraddadi et al. investigated the potential predictors of Middle East respiratory syndrome coronavirus (MERS-CoV) infection in subjects, discovering that diabetes and cardiovascular disease were all significantly associated with MERS-CoV disease [17]. Our research has shown that patients with underlying comorbidities are more susceptible to COVID-19 infection because their underlying diseases predispose them to infection and worsening state. This is supported by the findings of research conducted at other centers.

Limitations

It was a single-center study with severity assessed on the day of admission, which may underestimate further severity in different groups of patients. Second, the study evaluated specific risk factors that we considered. A more exhaustive review examining uncommon comorbid conditions such as connective tissue disorders, patients on biological agents, and genetic conditions causing immune compromise is required. Patients were not followed up for long-term survival, re-hospitalization, and mortality after discharge.

## Conclusions

Patients with multiple comorbidities had more severe COVID-19 compared to single morbidity. Patients with single comorbidity had more severe disease in comparison to those without any comorbidities. Male gender and age groups >50 years had a higher incidence of severe COVID-19. Comorbid conditions that are associated with severe COVID-19 include diabetes mellitus, obesity, CKD, heart disease, hypertension, and CLD. This will help us take early and active measures in this group of patients. We advocate for a low threshold for admission in this group. We can take precautionary measures of early hospitalization, initiate early treatment, and monitor vital oxygen requirements, thereby improving patient outcomes. Further evaluation and adequately powered prospective investigations are required to further evaluate the risk factors for severe COVID-19.
